# Predictive Value of the Loss of pRb Expression in the Malignant Transformation Risk of Oral Potentially Malignant Disorders: A Systematic Review and Meta-Analysis

**DOI:** 10.3390/cancers17020329

**Published:** 2025-01-20

**Authors:** María López-Ansio, Pablo Ramos-García, Miguel Ángel González-Moles

**Affiliations:** Biohealth Research Institute, IBS, School of Dentistry, University of Granada, 18071 Granada, Spain; lopansmar@correo.ugr.es

**Keywords:** pRb, retinoblastoma protein, oral potentially malignant disorders, oral leukoplakia, malignant transformation, oral cancer, systematic review, meta-analysis

## Abstract

Objective: The aim of this systematic review and meta-analysis was to qualitatively and quantitatively evaluate the current evidence on the significance of the loss of early stages of oral carcinogenesis in lesions diagnosed according to clinical and/or histopathological criteria and their evolution to oral cancer. Materials and Methods: We searched MEDLINE (through PubMed), Embase, Scopus and Web of Science for primary-level studies published before November 2024, designed as prospective or retrospective longitudinal cohorts, and not restricted by language or publication date. The risk of bias was critically assessed using the QUIPS tool. Meta-analyses, heterogeneity exploration, sensitivity and small-study effect analyses were conducted. Results: The inclusion criteria were met by six primary-level studies, which recruited 330 patients with OPMDs with follow-up data. The loss of pRb expression, assessed through immunohistochemistry, was significantly associated with a higher malignant transformation risk of OPMDs (RR = 1.92, 95%CI = 1.25–2.94, *p* = 0.003). The leukoplakia subgroup retained this significant association (*p* = 0.006), being the OPMD where the loss of pRb expression showed the best predictive value for malignant transformation (RR = 2.00, 95%CI = 1.22–3.29). Regarding the immunohistochemical technique and scoring methods, better performance and results were achieved by applying a cutoff point > 10% pRb-positive cells with nuclear staining (RR = 2.10, 95%CI = 1.30–3.38, 95%CI = 0.002). Conclussion: The present systematic review and meta-analysis supports that the loss of expression of the tumor suppressor pRb, assessed through immunohistochemistry, is a predictor of the malignant transformation risk of oral leukoplakias. Future studies are needed in other OPMDs following the recommendations provided based on current evidence gaps.

## Simple Summary

Oral cancer, a malignant neoplasm with invariable poor prognosis in the last 40 years (5-year survival rate of nearly 50%), accounts for a worldwide incidence of 377,713 new cases annually and 177,757 deaths per year. Oral cancer is usually preceded by oral potentially malignant disorders (OPMDs)—such as leukoplakia, oral lichen planus and oral submucous fibrosis—defined by the WHO Collaborating Centre for Oral Cancer as any mucosal abnormality that is associated with a statistical increased risk of developing oral cancer. Unfortunately, there are currently no tools available to accurately predict whether a patient in the early stages of oral carcinogenesis will develop oral cancer, being the presence and severity of epithelial dysplasia the histological marker universally applied for clinical practice. Nevertheless, this system presents relevant limitations related to imprecise and subjective assessments; therefore, emerging molecular biomarkers are under investigation. Among these biomarkers, retinoblastoma protein (pRb) is a well-recognized tumor suppressor in human oncogenesis, with roles linked to evading growth suppressors, a relevant hallmark of cancer. The loss of pRb expression regulates tumor initiation and early progression of neoplasms, and has been hypothesized to exert an oncogenic role during malignant transformation in early stages of oral carcinogenesis.

## 1. Introduction

Hanahan and Weinberg [1,2], in 2000 and 2011, communicated the distinctive identity hallmarks that allow for the classification of a cell in a neoplastic state, irrespective of its origin. The proposal included six canonical features (sustaining proliferative signaling, evading growth suppressors, resisting cell death, enabling replicative immortality, angiogenesis, activating invasion and metastasis), two enabling characteristics (genome instability and mutation, and promoting tumor inflammation) and two emergent characteristics (deregulating cellular energetics and evading immune destruction) [1,2]. The proposal by Hanahan and Weinberg [1,2] has achieved enormous repercussion and has favored the establishment of lines of research with the aim of evaluating the usefulness of these hallmarks in diagnostic, prognostic and therapeutic terms. Nevertheless, it should be recognized that there is a lack of information in the field of oral oncogenesis from its earliest stages—oral potentially malignant disorders (OPMDs)—to the later stages when the cancer is fully established [3].

The characteristics of cancer cells include the ability to evade growth-suppressive signals and resist cell death [1,2], which are essentially accomplished through the functions of the tumor suppressor genes RB and TP53, which encode the tumor suppressor protein pRb and p53, respectively [4]. Retinoblastoma protein (pRb) controls cell proliferation, arresting the G1 cell cycle phase, and stimulates differentiation and chromosomal stability [5,6]. This is essentially carried out through the sequestration of E2F transcription factors. The loss of the RB tumor suppressor role has a marked influence on tumor development [7]. The most representative evidence of the importance of loss of pRb function in tumor initiation has been obtained through the genetic study of family members with an inherited alteration in the RB gene alleles that predisposes them to the development of familial retinoblastoma [8,9,10]. It has also been shown in cervical and oropharyngeal cancer, closely associated with HPV infection, that these viruses inactivate pRb through E7 oncoprotein [11,12], and similar findings have been documented for virus-induced hepatocarcinoma [13]. These tumor-initiating actions linked to RB loss occur both in stem cells, where normofunctional RB keeps them quiescent, their usual state, and in mitotically inactivated differentiated cells, in which RB mutation allows these post-mitotic cells to reintegrate into the progenitor compartment, and especially in the proliferative pool of cells, which are called amplifying transitory cells in the oral epithelium, which constitute an intermediate step between stem cells and differentiated post-mitotic cells. There are indications that amplifying transitory cells could be a frequent origin of malignant and pre-malignant clones in the oral epithelium [14], where the loss of pRb expression could maintain proliferation by preventing their cell cycle exit in G1, which occurs physiologically in these cells after the development of several proliferative cycles [11,12,13,14,15].

Recently, several research groups have focused on the investigation of several molecular markers with translational potential for the prediction of the progression of early stages of oral carcinogenesis to oral cancer through secondary-level sources of evidence via systematic reviews and meta-analyses (such as aneuploidy [16], loss of heterozygosity [17], cyclin D1 [18], MMP9 [19], survivin [19], p16 [20], p53 [21], and podoplanin [22]) or through primary-level studies (such as antinuclear antibody [23]). Based on this background, these biomarkers are currently the most promising molecular tools in predicting malignant transformation in oral carcinogenesis.

Despite the aforementioned facts, we must recognize that, to date, there are no studies with evidence-based designs, in the form of systematic reviews and meta-analyses, that analyze the role of pRb protein alterations in the early stages of oral carcinogenesis. The main challenge in the management of oral cancer precursor lesions lies in the absence of reliable markers to predict which individual OPMDs will harbor a high risk of malignancy, which would enable the establishment of specific management for high-risk patients. The objective of the present systematic review and meta-analysis was to qualitatively and quantitatively evaluate the current evidence on the significance of the loss of pRb expression in the early stages of oral carcinogenesis, in lesions diagnosed according to clinical and/or histopathological criteria, and their evolution to oral cancer.

## 2. Materials and Methods

This systematic review and meta-analysis complied with the *MOOSE* and *PRISMA* reporting guidelines [24,25], and closely followed the criteria of *Cochrane Prognosis Methods Group* [26] and *Cochrane Handbook for Systematic Reviews of Interventions* [27].

### 2.1. Protocol

A detailed protocol outlining the methodology of this systematic review and meta-analysis was developed in advance and registered in the PROSPERO international prospective register of systematic reviews. This approach aimed to reduce bias and ensure greater transparency, precision, and integrity (www.crd.york.ac.uk/PROSPERO; registration code CRD42024614644; accessed on 26 November 2024). The protocol adhered to the PRISMA-P statement to ensure methodological rigor [28].

### 2.2. Search Strategy

The MEDLINE/PubMed, Embase, Scopus, and Web of Science databases were searched for studies published up to the upper date limit of November 2024, with no lower date limit applied. The search strategy was developed by combining thesaurus terms (e.g., MeSH or Emtree) with free-text terms (Table S1, Supplementary Materials) to maximize sensitivity. Additional screening was conducted by manually reviewing the reference lists of the included studies to identify further relevant studies. All references were managed using Mendeley v.1.19.8 (Elsevier, Amsterdam, The Netherlands), and duplicate references were removed with this software.

### 2.3. Eligibility Criteria

Inclusion criteria: (1) original studies with no restrictions on language or publication date; (2) assessment of loss of pRb expression using immunohistochemical techniques in samples from lesions in the early stages of oral carcinogenesis, diagnosed according to clinical and/or histopathological criteria; (3) analysis of malignant transformation risk, including data on progression and non-progression to oral cancer.; (4) longitudinal study design.

Exclusion criteria: (1) reviews, rejected articles, meta-analyses, case reports, letters, editorials, meeting abstracts, personal opinions, book chapters, or comments; (2) in vivo animal experimentation or in vitro laboratory; (3) evaluation of *RB* gene alterations (e.g., mutations or polymorphisms); (4) studies focused on OSCC without data on malignant transformation or precancerous; (5) observational cross-sectional studies or interventionist study design; (6) studies with insufficient statistical data to estimate relative risk (RR) with 95% confidence intervals (CI); (7) studies with overlapping populations, verified by reviewing author names, affiliations, patient sources, and recruitment periods.

### 2.4. Study Selection Process

Two authors (MLA and PRG) independently applied the eligibility criteria, resolving discrepancies by consensus. Articles were elected after two phases: first, the titles and abstracts of those apparently meeting the inclusion criteria were screened, and second, the authors engaged in full-text reading of the selected articles, excluding those that did not match the eligibility criteria. The evaluators were trained and calibrated to identify potentially included studies, conducting an assessment round (50 papers each). A final inter-rater agreement score was calculated using Cohen’s kappa statistic [29], achieving an almost perfect agreement (99.95% of agreement; kappa value = 0.91).

### 2.5. Data Extraction

Datasets were extracted from the included articles, documenting the information in a standardized data collection form using Excel (v.16/2018, Microsoft, Redmond, WA, USA). Discrepancies were resolved through consensus. Statistical data, including interquartile ranges, medians, and/or minimum–maximum values, were recalculated and converted into means ± standard deviation (SD) using the methodologies proposed by Luo et al. (2018) and Wan et al. (2014) [30,31]. When appropriate, data from two or more subgroups expressed as means ± SD were combined into a single dataset using the formula provided in the Cochrane Handbook [27]. The variables extracted from each article included the first author, publication year, country and continent, publication language, study design, recruitment and follow-up periods, sample size, anatomical site, patient demographics (sex and age), tobacco and alcohol consumption, diagnostic criteria of early precursor lesions (clinical and/or histopathological), data on oral epithelial dysplasia and oral cancer development, immunohistochemical methods (e.g., anti-pRb antibody specifications such as dilution, incubation time, and temperature), cutoff point, scoring system, and the proportion of cases exhibiting loss of pRb expression.

### 2.6. Appraisal of Quality and Risk of Bias

Two authors (MLA and PRG) conducted a critical appraisal of the quality and risk of bias in primary-level studies using the Quality in Prognosis Studies (QUIPS) tool, developed by the *Cochrane Prognosis Methods Group* [32]. Six potential bias domains were evaluated: (1) study participation, (2) study attrition, (3) prognostic factor measurement, (4) outcome measurement, (5) study confounding, and (6) statistical analysis and reporting. The risk of bias for each domain was classified as high, moderate, or low. Subsequently, an overall risk of bias score was determined based on a methodology previously established by our research group [33]. In summary, each study was assigned an overall risk of bias rating—either low or high—derived primarily from the evaluation of domains 3 and 5, which were identified as critical domains. These assessments were used to statistically analyze the impact of methodological quality in primary-level studies on the meta-analytical findings.

### 2.7. Statistical Analysis

The loss of pRb expression was assessed in accordance with the cutoff values designed and specified by primary-level studies. Relative risks (RRs) with 95% confidence intervals (CIs) were calculated to evaluate the risk of malignant transformation of lesions in the aearly stages of oral carcinogenesis, diagnosed according to clinical or histopathological criteria, in patients exhibiting a loss of pRb expression. These calculations were performed using the inverse-variance method within a random-effect model, based on the DerSimonian and Laird methodology. This statistical model was selected to account for potential sources of methodological, clinical and statistical heterogeneity across study subpopulations (presumably including differences among OPMDs, immunohistochemical techniques for detecting pRb expression, e.g., differences among anti-pRb antibodies, and affected oral subsites). When the RRs were evaluated in both univariate and multivariate models, the effect size was directly extracted and computed from the multivariate model, which reflects a greater adjustment for potentially confounding variables. If RR data were not reported, hazard ratios (HRs) or odds ratios (ORs) were extracted as an approximation of this measure. Forest plots were built for all meta-analyses to graphically represent the overall effect.

Heterogeneity across studies was assessed using the χ^2^-based Cochran’s Q test. Due to the limited statistical power of this test, a *p*-value < 0.10 was considered indicative of significant heterogeneity. Additionally, the Higgins’ I^2^ statistic was employed to quantify the proportion of variance in observed effects attributable to true effect variability rather than sampling error. I^2^ values between 50% and 75% were interpreted as indicative of a moderate-to-high degree of inconsistency among studies [34,35]. Preplanned subgroup meta-analyses were performed to explore potential sources of heterogeneity.

Secondary analyses were conducted to evaluate the stability and reliability of the meta-analytical results. Sensitivity analyses were performed to assess the influence of individual primary-level studies on the pooled estimate [36], using the “leave-one-out” method, which involves sequentially repeating the meta-analysis while omitting one study at a time. Additionally, small-study effects analyses were undertaken to detect potential biases, including publication bias. Funnel plots were constructed, and the Egger regression test was applied, which involves a linear regression of the effect estimates against their standard errors, weighted by 1/[variance of the effect estimate]. A *p*-value for the Egger test (pEgger) < 0.10 was considered indicative of significant small-study effects [37]. All statistical analyses were performed using Stata software (v.16.1, Stata Corp, College Station, TX, USA).

## 3. Results

### 3.1. Results of the Literature Search

**Figure 1:**
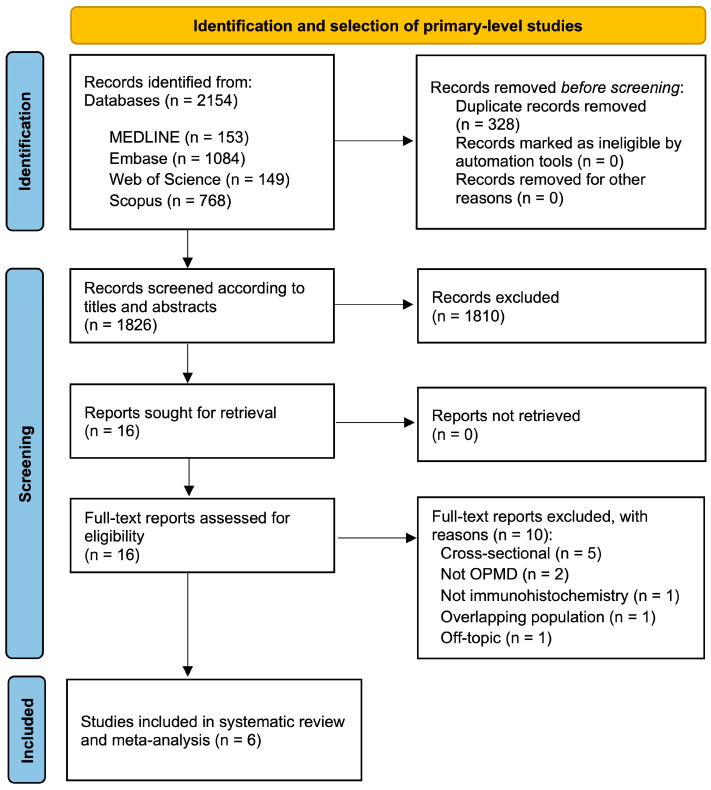
Flow diagram of the process of identification and selection of primary-level studies offering scientific information on the loss of pRb expression and oral potentially malignant disorders malignant transformation risk.

The flow diagram presented in Figure 1 outlines the process of study identification and selection. A total of 2154 records were retrieved: 1084 from Embase, 768 from Scopus, 153 from MEDLINE/PubMed, and 149 from Web of Science. No further records could be identified by hand-searching the reference lists of the retrieved studies. Following the removal of duplicates, 1826 studies were screened based on their titles and abstracts. Of these, 16 articles were deemed potentially eligible and selected for full-text systematic review. However, 10 articles did not meet all the eligibility criteria; the excluded studies and their respective exclusion reasons are detailed in List S1 (Supplementary Materials). Ultimately, six primary-level studies were included in the final sample for qualitative and quantitative analyses [38,39,40,41,42,43].

### 3.2. Study Characteristics

Table 1 summarizes the key characteristics of the selected studies, while Table S2, in the Supplementary Materials, provides a more detailed overview of the characteristics of each primary-level study. The sample size is composed of a total of six studies, published between 1998 and 2011, including 330 patients with OPMDs and data on malignant transformation. The number of patients ranged from 9 to 113 patients. Three studies categorized OPMDs among clinical lesions (leukoplakias and proliferative verrucous leukoplakias), while three other studies differentiated histopathological lesions (keratosis, hyperplasia, and presence/absence of epithelial dysplasia). The data were heterogenous in clinical and histopathological terms, and some studies report mixed OPMDs, which can only be described through a narrative synthesis, but not differentiated at the meta-analytic level due to the lack of individual patient data. Specifically, Schoelch et al. (1998) [39] analyze 18 cases through histological criteria and refer to focal keratosis, mild dysplasia, and moderate dysplasia. Girod et al. (1998) [38] analyze 113 cases of patients with oral leukoplakia or oral lichen planus, documented according to the guidelines of the DOSAK (German-Austrian-Swiss Association for Head and Neck Tumours). Nevertheless, data on malignant transformation are only reported according to histopathological findings, and it is also unknown whether the malignant transformation cases correspond to lichen planus or to leukoplakia. Ghazali et al. (2003) [40] described nine cases of proliferative verrucous leukoplakia according to Hansen’s diagnostic criteria. It should also be noted that two patients were also associated with oral submucous fibrosis. Soni et al. (2005) [41] conducted a prospective cohort on 90 patients with oral leukoplakias, with histological evidence of oral epithelial hyperplasia or dysplasia. Shah et al. (2007) [42] published 70 cases with a clinical diagnosis of oral submucous fibrosis or oral leukoplakias; however, the data and analyses reported were actually histopathological lesions with hyperplasia or dysplasia and their progression to oral cancer. Finally, Nasser et al. (2011) [43] analyzed 40 cases of oral leukoplakias without epithelial dysplasia but did not report the specific clinical diagnostic criteria.

On the other hand, in relation to the anti-pRb antibodies used for immunohistochemical technique, a wide spectrum was applied by the authors of the primary-level studies (clone IF-8, by two studies, and Ab-1, pRb and Rb-1 clones by one study, while a single study did not report the anti-pRb antibody). These antibodies were applied in dilutions ≤ 1:50 across 4 studies, 1:100 in one study, and not reported by a single study. Furthermore, three studies incubated their antibodies overnight, and one study incubated these for 1 h, while two studies did not report it. On the other hand, the incubation temperature was 4 °C in three studies and not reported in three other studies. Geographically, two studies were conducted in Europe, three in Asia, and one in North America.

### 3.3. Qualitative Evaluation

**Figure 2:**
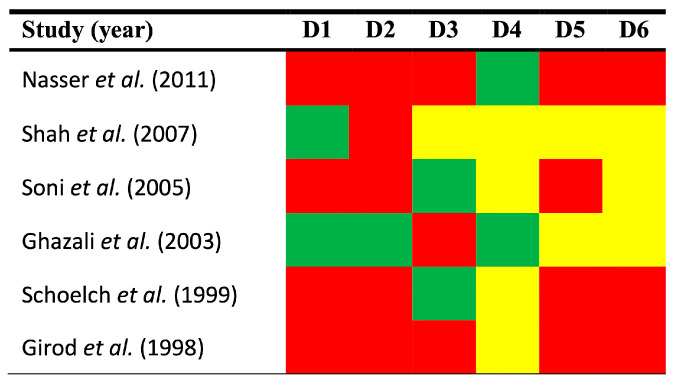
Quality plot graphically depicting the methodological quality and potential risk of bias across primary-level studies [38,39,40,41,42,43], critically verified by applying the QUIPS tool, developed by the Cochrane Prognosis Methods Group, which considers the following domains: (D1) study participation, (D2) study attrition, (D3) prognostic factor measurement, (D4) outcome measurement, (D5) study confounding, and (D6) statistical analysis and reporting. Risk of bias was qualified as low (green), moderate (yellow), or high (red) for each domain.

The qualitative analysis was performed using the Quality in Prognosis Studies-QUIPS tool, which evaluates potential sources of bias across six domains (Figure 2).

*Study participation.* The risk of bias in this domain was low in 33.33% of the reviewed studies and high in 66.67%. The most common sources of bias included the failure to report the grade of oral epithelial dysplasia and the recruitment periods, among other clinicodemographical characteristics of patients.

Study attrition. The risk of bias in this domain was low in 16.67% of the reviewed studies and high in 83.33% (Figure 2). Although all studies were designed as longitudinal cohorts with follow-up data, some studies did not report essential datasets (e.g., average months, as means ± standard deviation or medians with interquartile range or minimum-maximum values). Furthermore, no study reported the dropout rate of patients with precision, the intention to gather information and reasons for patients lost to follow-up, or the description of their characteristics, essential to assess differences between the characteristics of the baseline and final study samples.

*Prognostic factor measurement*. The risk of bias in this domain was low in 33.33% of the reviewed studies, moderate in 16.67%, and high in 50.00% (Figure 2). The most significant sources of bias were the lack of sufficient information regarding the cutoff point design, the immunohistochemical technique, and the scoring system used to assess pRb expression. It is important to note that no significant biases were identified concerning the use of optimized cutoff points based on data analysis [44], which could be one of the most critical sources of bias in this domain. Finally, a single primary-level study failed to report essential data regarding the use of the anti-pRb antibody. This is a very relevant factor because the difference in the type of anti-pRb antibody affects the results of the immunohistochemical technique and could act as an effect modifier variable in the interpretation of the expression of this protein.

Outcome measurement. The risk of this bias was low in 33.33%, and moderate in 66.67% (Figure 2) of the studies. This domain received the best score, as the clinical and histopathological methods used to diagnose OSCC development are universally accepted. Furthermore, the lack of information regarding the system employed was not considered indicative of low quality or a potential risk of bias.

*Study confounding*. The risk of this bias was moderate in 33.33% of the reviewed studies and high in 66.67%. The most common potential sources of bias were the failure to account for confounders in the study design or to measure all relevant confounders. No study explicitly defined *a priori* the factors considered potential confounders, nor did any study subsequently discuss the potential biological interactions between these factors and the loss of pRb expression.

*Statistical analysis and reporting.* The risk of this bias was considered to be moderate in 50.00% of the reviewed articles and high in 50.00%. The most common biases were selective outcome reporting and the failure to estimate relative risks with their corresponding 95% CI despite the fact that these statistics provide more informative insights than simple *p*-values (i.e., in terms of magnitude, direction and precision of effect size).

### 3.4. Quantitative Evaluation (Meta-Analysis)

The main quantitative results of the present meta-analytical study are reported in Table 2 and in forest plots (Figure 3 and Figures S1–S9, in the Supplementary Materials).

**Figure 3:**
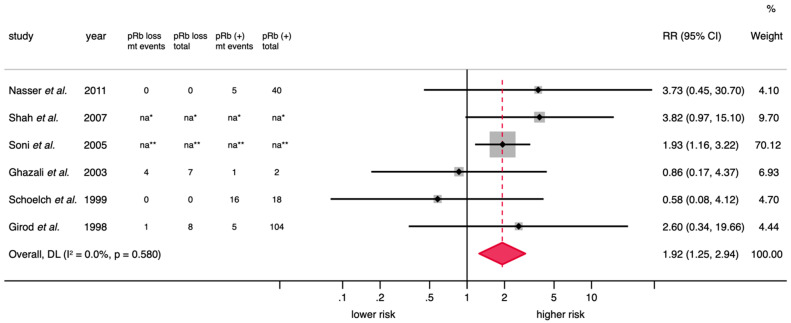
Forest plot graphically representing the meta-analysis on the association between the loss of pRb expression and malignant transformation. An RR > 1 suggests that the loss of pRb expression is associated with a higher malignant transformation risk. Diamonds indicate the pooled RR with their corresponding 95% CIs. Six primary-level studies were included in this meta-analysis [38,39,40,41,42,43]. RR, relative risk; CIs, confidence intervals; mt, malignant transformation; na, not applicable; DerSimonian and Laird, DL. Random-effect model, inverse-variance weighting based on the DL method; * Effect size was directly extracted and computed from multivariate regression analysis; ** Effect size was directly extracted and computed from univariate regression analysis.

*Meta-analysis on the association between the loss of pRb expression and malignant transformation risk*. A random-effect model estimated a significantly increased malignant transformation risk among patients in the early stages of oral carcinogenesis and loss of pRb expression (RR = 1.92, 95% CI = 1.25–2.94, *p* = 0.003). Primary-level studies showed consistent results and statistical heterogeneity was not significant (*p* = 0.58, I^2^ = 0.0%; Figure 3, Table 2).

*Subgroups meta-analysis*. Several of the meta-analyzed subgroups preserved the precedent significant association, stratified by geographical area (Asia: RR = 1.95, 95% CI = 1.23–3.09, *p* = 0.004); by type of diagnostic criteria (oral leukoplakia among clinical lesions: RR = 2.00, 95% CI = 1.22–3.29, *p* = 0.006); by immunohistochemical pattern (nuclear pattern: RR = 1.86, 95% CI = 1.20–2.88, *p* = 0.005); by immunohistochemical anti-pRb antibody (IF-8: RR = 1.93, 95% CI = 1.16–3.22, *p* = 0.01); by antibody dilution (1/100: RR = 1.93, 95% CI = 1.16–3.22, *p* = 0.01); by cutoff point for loss of pRb expression (≤10%: RR = 2.10, 95% CI = 1.30–3.38, *p* = 0.002); and by methodological quality (low risk of bias subgroup: RR = 1.95, 95% CI = 1.04–3.64, *p* = 0.04) (Table 2 and Figures S1–S9, Supplementary Materials).

### 3.5. Quantitative Secondary Analyses

*Sensitivity analysis*. The overall results did not substantially vary after the sequential repetition of meta-analyses, omitting one study each time (“leave-one-out” method) (Figure S11, Supplementary Materials). This suggests that the pooled relative risk reported does not depend on the influence of a particular individual primary-level study. It should be noted that according to the selection and the results, only two articles were referred to for the meta-analysis of leukoplakia, although one of them provides 70.12% of the weight of all leukoplakias (vs. 74.22% of leukoplakia and of the entire sample); it was carried out based on histological criteria, hyperplasia/dysplasia. Furthermore, in the study by Soni et al. 2005, there are marked differences between the univariate and multivariate analysis.

*Analysis of small-study effects*. Visual inspection analysis of the funnel plot showed no asymmetry (Figure S10, Supplementary Materials), confirmed by the statistical test (p_Egger_ = 0.86). Therefore, the presence of small-study effects—such as publication bias—could be potentially ruled out, reaffirming the reliability of meta-analytical results.

## 4. Discussion

Our meta-analysis on the predictive value of the loss of expression of the pRb tumor suppressor protein, carried out on a total of 6 studies and 330 patients in the early stages of oral carcinogenesis, shows that the loss of pRb expression assessed by immunohistochemical techniques behaves as a significant risk marker for progression to oral cancer in oral leukoplakia (RR = 2.00, 95% CI = 1.22–3.29, *p* = 0.006). This result, derived from 130 patients, is robust, as reflected in its narrow confidence interval. Primary-level studies conducted on the subject included OPMDs, diagnosed as clinical lesions, such as oral leukoplakia or proliferative verrucous leukoplakia (PVL), or histopathological lesions. Only one study, including nine patients, dealt with pRb analysis in PVL [40]; so, no conclusions can be drawn at this moment about the oncogenic implications of this protein in this OPMD. Three additional studies (191 patients) [38,39,42] considered a mix of aggregated premalignant lesions (e.g., oral lichen planus or oral submucous fibrosis) and actually performed their analyses on histopathological lesions, such as oral epithelial hyperplasia or dysplasia, and their evolution to oral cancer. Furthermore, these studies did not provide independent malignant transformation data for each of the OPMDs investigated. The loss of pRb expression was not shown to behave as a predictor of progression to oral cancer in this heterogenous subgroup of histopathological lesions (RR = 2.09, 95% CI = 0.70–6.28, *p* = 0.19). Thus, this analysis offers results with limited application to clinical practice. In our opinion, this result could reflect that the loss of pRb would only behave as a predictor of cancer risk in oral leukoplakia, as discussed above, whereas in the other OPMDs, the interpretation is uncertain due to the small sample size and clinical heterogeneity, and it could be insufficient to elucidate whether the lack of significance derives from a lack of relevance of pRb in their malignant transformation or if it is indicative of a lack of statistical power. It is evident that there is a need for further studies on pRb in OPMDs other than oral leukoplakia [45].

The loss of pRb expression in normal tissues could not be investigated in the present systematic review and meta-analysis, but understanding the implications and relative frequency of this oncogenic event in healthy oral mucosa is important and should be considered as a line of future research. Soni et al. (2005) [41] analyzed a comparator group of 81 normal mucosa cases, showing a pRb expression negative rate of 47%, higher than that of precancerous lesions (33%). Unfortunately, 70 out of the 81 cases (86.42%) were obtained from cancerous lesions (i.e., oral epithelium distant or close to oral carcinomas, with histologic evidence of normal epithelium). The consideration of matched morphologically normal specimens from surgical margins is universally considered an inadequate control group, severely biased and not suitable for measuring pRb expression. This is due to the fact that the presence of genetically altered premalignant fields is well established and accepted in cancers arising in the head and neck region [46]. The field cancerization theory highlights that oral mucosa apparently clinically healthy harbors early oncogenic molecular alterations, with very important prognostic implications, and it is responsible for up to 30% of patients subsequently developing second tumors in neighboring anatomical areas of the upper aerodigestive tract within 5 years [47,48]. However, the lack of primary-level studies published to date should also be considered a relevant evidence gap; so, we advise that future studies investigate the loss of pRb expression in control groups adequately obtained from normal oral mucosa of healthy patients.

As commented in the Section 1, it has also been shown in cervical and oropharyngeal cancer, closely associated with HPV infection, that these viruses inactivate pRb through E7 oncoprotein [11,12]. Consequently, during the design and conduction of the present systematic review and meta-analysis, we also attempted to investigate the relationship between the status of HPV infection and the loss of pRb expression in OPMDs. Unfortunately, the existing body of evidence did not allow us to perform a statistical analysis using meta-analytic techniques due to the fact that only two longitudinal studies have been published on this topic to date and both offer very heterogeneous results in relation to the method of HPV analysis. Ghazali et al. [40] investigated the implications of the protein pRb and HPV status in Malaysian patients affected by proliferative verrucous leukoplakia (PVL). Due to the low prevalence of this OPMD, only nine patients were analyzed. In this cohort, only two patients harbored a positive-HPV status, and both patients overexpressed pRb protein according to the immunohistochemical analysis. However, the sample size is too low to draw any conclusions from this primary-level study. A second study conducted by Nasser and colleagues [43] analyzed the loss of pRb expression in German patients affected by oral leukoplakias. This study cohort furthermore tried to explore HPV status through the immunohistochemical analysis of the p16^INK4a^ protein. Nevertheless, none of the patients showed reduced pRb staining concomitant with p16^INK4a^ protein overexpression. So, the authors concluded that HPV does not play an important role in pRb down-regulation in oral leukoplakias. Therefore, the evidence derived from the two primary-level studies published to date does not allow us to conclude anything in this regard. However, the present systematic review and meta-analysis, based on this background, recommends that future primary-level studies investigate the relationship between HPV infection status, the loss of pRb expression and the risk of malignant transformation of oral potentially malignant disorders.

Our meta-analysis shows that the loss of nuclear pRb expression was associated with the predictive value (RR = 1.86, 95% CI = 1.20–2.88, *p* = 0.005), reflecting that its actions are linked to its ability to inhibit E2F transcription factors in the cell nucleus. We also found that the ideal cutoff point for the consideration of a case as a loss of expression was ≤10% of pRb-positive cells (RR = 2.10, 95% CI = 1.30–3.38, *p* = 0.002). It should also be noted that the range of the percentage of loss of pRb expression was very wide, from 0% to 33.33% in patients with oral leukoplakia, and it was 77.78% in cases of patients with proliferative verrucous leukoplakia. Future studies are also needed to better understand these variations in the presence/loss of protein expression. Likewise, the monoclonal antibody that performed best in the immunohistochemical technique was IF-8 (RR = 1.93, 95% CI = 1.16–3.22, *p* = 0.01), which also occurred in our previous meta-analysis on the prognostic value of pRb in oral cancer [49]. We also found that the higher methodological quality of the primary-level studies included in the meta-analysis was associated with a better predictive performance for the loss of pRb expression (RR = 1.95, 95% CI = 1.04–3.64, *p* = 0.04). The meticulous analysis of immunohistochemistry should also be considered with caution given the evolution that these techniques have experienced in the last 10–25 years. Consequently, recommendations for future research studies on the subject include an adequate description of the study sample, prolonged follow-up of patients with OPMDs, transparent and robust clinical and histopathological diagnostic criteria, adequate description of the immunohistochemical technique, control of confounding factors—e.g., tobacco use—and the application of appropriate statistical methods, as well as the avoidance of selective reporting bias. It is also interesting to note that, in our previous meta-analysis on the prognostic value of loss of pRb expression in oral cancer [49], it was found that the loss of pRb expression in oral cancer did not negatively influence patient prognosis. In comparison to our results in cancer, the fact that our current results indicate that the loss of pRb is important in the malignancy of oral leukoplakia may reflect that this tumor suppressor protein is relevant in the early stages of oral carcinogenesis but not in the later stages linked to tumor progression and extension. Interestingly, all immunohistochemical scores of cases derive from baseline reports, i.e., these primary-level studies usually report overexpression or loss of pRb protein at initial diagnosis as a predictive tool for clinicopathological evolution of the case. But it would be interesting to know if there are changes in the up-regulation or down-regulation of the protein. Although this is the traditional way of performing this type of analysis, it could be more informative to also know the changes in pRb staining as a lesion evolves through the different steps of oral carcinogenesis over time.

As potential limitations that should be discussed, heterogeneity is a common concern in most systematic reviews and meta-analyses. In this sense, the most important limitation of the present meta-analysis should be attributed to the clinical and methodological heterogeneity arising from the discrepancy of the clinical–histological inclusion criteria, as well as the reporting of mixed OPMDs, which could not be differentiated and analyzed independently due to the primary-level studies’ failure to report individual participant data, decreasing the clinical applicability of the present meta-analysis. Another limitation of the present study is the diversity of different anti-pRb antibodies that were applied in the immunohistochemical technique across primary-level studies. We consider that the difference in the type of anti-pRb antibody affects the results of the immunohistochemical technique and should therefore be understood as a considerable source of methodological heterogeneity in the present meta-analysis. In this sense, the interpretation of the results of this meta-analysis should be undertaken with caution. We recommend that future studies analyze in depth the implications of the different anti-pRb antibodies, particularly the IF-8 clone, which showed significant differences (RR = 1.93, 95% = 1.16–3.22, *p* = 0.01), although its results were heterogeneous, showing an I-squared higher than 50%, and derived from a small-sample-size subgroup (n = 2). On the other hand, several studies did not report detailed datasets for the parameters of interest (e.g., aggregated data on clinical lesions not stratified by type of OPMD in an independent manner, as well as limited information from clinicodemographical parameters such as age, sex, tobacco and alcohol use, and presence and grade of oral epithelial dysplasia, among others), restricting the implementation of secondary analysis. As another recommendation for future investigation, primary-level studies should generally improve the methodological rigor at the level of reporting. As a major strength that should be highlighted, all the primary-level studies analyzed are longitudinal cohorts which followed their patients over time, offering a higher quality of evidence in contrast to the majority of studies published for the study of other biomarkers in OPMDs, which are of a cross-sectional nature. Therefore, this meta-analysis offers a more accurate assessment of causality and risk analysis, and in this sense, may offer conclusions closer to reality.

## 5. Conclusions

In conclusion, the loss of expression of the tumor suppressor protein pRb, evaluated by immunohistochemical methods, behaves as a risk marker for cancer progression in oral leukoplakia, a relevant and well-known oral potentially malignant disorder. Further research on the predictive value of pRb loss in other oral potentially malignant disorders is needed following the recommendations provided based on the current evidence gaps.

## Figures and Tables

**Table 1:** Summarized characteristics of the study sample.

Total	6 Studies
Year of publication	1998–2011
Total patients (range)	330 (9–113)
Diagnostic criteria	
Clinical lesions	
Leukoplakia	2 studies
Proliferative verrucous leukoplakia	1 study
Histopathological lesions	
Keratosis, Hyperplasia, Dysplasia	3 studies
pRb immunohistochemical pattern	
Nuclear staining	5 studies
Not reported	1 study
Anti-pRb antibody	
pRb	1 study
Rb-1	2 studies
IF-8	1 study
Ab-1	1 study
Not reported	1 study
Anti-pRb antibody dilution	
≤1:50	4 studies
1:100	1 study
Not reported	1 study
Anti-pRb antibody incubation time	
Overnight	3 studies
1 h	1 study
Not reported	2 studies
Anti-pRb antibody incubation temperature	
4 °C	3 studies
Not reported	3 studies
Cutoff point for pRb overexpression	
1%	2 studies
10%	2 studies
Not reported	2 studies
Study design	
Retrospective cohorts	6 studies
Geographical region	
Asia	3 studies
Europe	2 studies
North America	1 study
Total	3 continents
Table S2 (Supplementary Materials) describes in detail the characteristics of the studies.	

**Table 2:** Predictive value of the loss of pRb expression on the malignant transformation risk of OPMDs.

Meta-Analyses	No. of Studies	No. of Patients	Stat. Model	Wt	Pooled Data		Heterogeneity	
					RR (95% CI)	*p*-Value	*P_het_*	*I^2^* (%)
Loss of pRb expression and malignant transformation risk (all) ^a^	6	330	REM	D-L	1.92 (1.25–2.94)	0.003	0.58	0.0
Subgroup analysis by geographical region ^b^								
Asia	3	159	REM	D-L	1.95 (1.23–3.09)	0.004	0.39	0.0
Europe	2	153	REM	D-L	3.09 (0.72–13.35)	0.13	0.81	0.0
North America	1	18	—	—	0.58 (0.08–4.16)	0.59	—	0.0
Subgroup analysis by type of diagnostic criteria ^b^								
Oral leukoplakia	2	130	REM	D-L	2.00 (1.22–3.29)	0.006	0.55	0.0
Proliferative verrucous leukoplakia	1	9	—	—	0.86 (0.17–4.36)	0.86	—	0.0
Keratosis, hyperplasia or dysplasia	3	191	REM	D-L	2.09 (0.70–6.28)	0.19	0.30	16.8
Subgroup analysis by immunohistochemical pattern ^b^								
Nuclear	5	290	REM	D-L	1.86 (1.20–2.88)	0.005	0.50	0.0
Not reported	1	40	—	—	3.73 (0.45–30.81)	0.22	—	0.0
Subgroup analysis by anti-pRb antibody ^b^								
pRb	1	40	—	—	3.73 (0.45–30.81)	0.22	—	0.0
Rb-1	2	78	REM	D-L	1.71 (0.27–10.62)	0.57	0.12	57.7
IF-8	1	90	—	—	1.93 (1.16–3.22)	0.01	—	0.0
Ab-1	1	113	—	—	2.60 (0.34–19.77)	0.36	—	0.0
Not reported	1	9	—	—	0.86 (0.17–4.36)	0.86	—	0.0
Subgroup analysis by anti-pRb antibody dilution ^b^								
≤1:50	4	127	REM	D-L	1.74 (0.67–4.51)	0.25	0.58	0.0
1:100	1	90	—	—	1.93 (1.16–3.22)	0.01	—	0.0
Not reported	1	113	—	—	2.60 (0.34–19.77)	0.36	—	0.0
Subgroup analysis by anti-pRb antibody incubation time ^b^								
Overnight	3	109	REM	D-L	2.30 (0.87–6.10)	0.09	0.34	6.4
1 h	1	18	—	—	0.58 (0.08–4.16)	0.59	—	0.0
Not reported	2	203	REM	D-L	1.96 (1.20–3.22)	0.008	0.78	0.0
Subgroup analysis by anti-pRb antibody incubation temperature ^b^								
4 °C	3	109	REM	D-L	2.30 (0.87–6.10)	0.09	0.34	6.4
Not reported	3	221	REM	D-L	1.83 (1.13–2.95)	0.01	0.48	0.0
Subgroup analysis by cutoff point for pRb protein overexpression ^b^								
1%	2	131	REM	D-L	1.20 (0.28–5.23)	0.80	0.30	7.5
10%	2	150	REM	D-L	2.10 (1.30–3.38)	0.002	0.36	0.0
Not reported	2	49	REM	D-L	1.52 (0.37–6.19)	0.56	0.28	14.2
Subgroup analysis by overall risk of bias in primary-level studies ^b^								
Low RoB	3	168	REM	D-L	1.95 (1.04–3.64)	0.04	0.31	15.9
High RoB	3	162	REM	D-L	1.74 (0.59–5.17)	0.32	0.50	0.0

Abbreviations: Stat., statistical; Wt, method of weighting; RR, relative risk; CIs, confidence intervals; REM, random-effect model; D-L, DerSimonian and Laird method; OPMDs, oral potentially malignant disorders; RoB, risk of bias. a—Meta-analysis of aggregate (summary) data. b—Subgroup meta-analyses

## Data Availability

The data that support the findings of this study are available in the Supplementary Materials of this article.

## Supplementary Materials

The following supporting information can be downloaded at: www.mdpi.com/article/10.3390/cancers17020329/s1, Table S1. Search strategy for each database, number of results, and execution date; Table S2. Characteristics of the included studies (n = 6); Figure S1. Forest plot graphically representing the stratified meta-analysis on the association between the loss of pRb expression and the malignant transformation risk of OPMDs by geographical region; Figure S2. Forest plot graphically representing the stratified meta-analysis by type of diagnostic criteria (i.e., clinical lesions vs. histopathological lesions); Figure S3. Forest plot graphically representing the stratified meta-analysis on the association between the loss of pRb expression and the malignant transformation risk of OPMDs by immunohistochemical pattern; Figure S4. Forest plot graphically representing the stratified meta-analysis on the association between the loss of pRb expression and the malignant transformation risk of OPMDs by anti-pRb antibody; Figure S5. Forest plot graphically representing the stratified meta-analysis on the association between the loss of pRb expression and the malignant transformation risk of OPMDs by anti-pRb antibody dilution; Figure S6. Forest plot graphically representing the stratified meta-analysis on the association between the loss of pRb expression and the malignant transformation risk of OPMDs by anti-pRb antibody incubation time; Figure S7. Forest plot graphically representing the stratified meta-analysis on the association between the loss of pRb expression and the malignant transformation risk of OPMDs by anti-pRb antibody incubation temperature; Figure S8. Forest plot graphically representing the stratified meta-analysis on the association between the loss of pRb expression and the malignant transformation risk of OPMDs by cutoff point for pRb overexpression; Figure S9. Forest plot graphically representing the stratified meta-analysis on the association between the loss of pRb expression and the malignant transformation risk of OPMDs by overall risk of bias in primary-level studies; Figure S10. A funnel plot of estimated logRRs against their standard errors, graphically representing the analysis of small-study effects on the association between the loss of pRb expression and OPMDs malignant transformation risk; Figure S11. Interval plot graphically representing the sensitivity analysis of the studies pooled in the meta-analysis on the association between the loss of pRb expression and OPMDs malignant transformation risk; List S1. List of full-text excluded studies, with reasons.
