# Supplementary material for: Predictive Value of the Loss of pRb Expression in the Malignant Transformation Risk of Oral Potentially Malignant Disorders: A Systematic Review and Meta-Analysis
Source: Cancers (Basel). 2025 Jan 20;17(2):329. doi: 10.3390/cancers17020329 (PMC11764026; doi:10.3390/cancers17020329)
Supplement: Supplementary file 1 [file cancers-17-00329-s001.zip › cancers-3403275-supplementary.pdf]

Supplementary information to the manuscript

Predictive value of the loss of pRb expression of the malignant transformation risk of oral potentially malignant disorders: a systematic review and meta-analysis

Table of contents

1. Search strategy....3

2. Descriptive characteristics of the study sample....4

3. Meta-analyses on the association between the loss of pRb expression and the malignant transformation risk of OPMDs....5

4. Analysis of small-study effects. 14

5. Sensitivity analysis....15

6. List of excluded studies with reasons....16

1. Search strategy

Table S1. Search strategy for each database, number of results, and execution date.

Database	Query/Search Strategy	Results/ Items found	Search time limits
MEDLINE	("Retinoblastoma[Mesh Terms]" OR “retinoblastoma”[All Fields] OR “rb”[All Fields] OR “prb”[All Fields] OR “OSRC”[All Fields] OR “pp110”[All Fields] OR “p105-Rb”[All Fields] OR “PPP1R130”[All Fields] OR “p110-RB1”[All Fields]) AND ("mouth"[MeSH Terms] OR "mouth"[All Fields] OR "oral"[All Fields] OR oropharyn*[All Fields]) AND ("carcinoma, squamous cell"[MeSH Terms] OR ("carcinoma"[All Fields] AND "squamous"[All Fields] AND "cell"[All Fields]) OR "squamous cell carcinoma"[All Fields] OR “dysplasia"[All Fields] OR “potentially malignant disorders”[All Fields] OR premalign*[All Fields] OR precancer*[All Fields] OR “leukoplakia”[All Fields] OR “erythroplakia”[All Fields] OR “lichen planus”[All Fields] OR “submucous fibrosis”[All Fields]) AND (“transformation”[All Fields] OR “progression”[All Fields] OR “risk”[All Fields])	153	Nov-2024
Embase	('retinoblastoma'/exp OR 'rb' OR 'prb' OR 'osrc' OR 'pp110' OR 'p105-Rb' OR 'ppp1r130' OR 'p110-rb1') AND ('squamous cell carcinoma'/exp OR 'squamous cell carcinoma' OR ('squamous' AND ('cell'/exp OR 'cell') AND ('carcinoma'/exp OR 'carcinoma')) OR 'dysplasia' OR 'potentially malignant disorders' OR 'premalign*' OR 'precancer'/exp OR 'precancer*' OR 'leukoplakia'/exp OR 'leukoplakia' OR 'erythroplakia' OR 'erythroplakia' OR 'lichen planus'/exp OR 'lichen planus' OR 'submucous fibrosis') AND ('transformation' OR 'progression' OR 'risk')	1,084	Nov-2024
Web of Science	TS=("retinoblastoma" OR "rb" OR “prb" OR “osrc” OR “pp110” OR “p105-Rb” OR “ppp1r130” OR “p110-rb12”) AND TS=(mouth OR oral OR oropharyn*) AND TS=(“squamous cell carcinoma”) AND TS=(transformation OR progression OR risk)	149	Nov-2024
Scopus	TITLE-ABS-KEY(("retinoblastoma" OR "rb" OR “prb" OR “osrc” OR “pp110” OR “p105-Rb” OR “ppp1r130” OR “p110-rb12”) AND (“mouth” OR “oral”) AND (“squamous cell carcinoma” OR neoplas* or “cancer”))	768	Nov-2024
Total			2,154

2. Table S2 .Characteristics of the included studies (n=6)

Study (year)	Country	Publication language	Study design (recruitment period)	Follow up, m, mean±SD	Patients with precancerous status and progression to cancer								Analysis of pRb protein expression				
					Sample Size, n	Sex, M(%)/F, n; age, y (mean±SD)	Tobacco, n (%)	Alcohol, n (%)	Affected oral subsites	Clinical lesions (diagnostic criteria)	Histopathological Lesions (Histological criteria)	Cancer development, n	Methods	Anti-pRb antibody (dilution, incubation time, temperature)	IHC patern	IHQ cutoff point (%)	Loss of pRb n (%)
Nasser *et al.* (2011)	Germany	English	Retrospective cohort (1993-2006)	median= 108	40	NR (NR)	NR	NR	NR	Oral leukoplakia (NR)	Absence of oral epithelial dysplasia (NR)	5	IHQ	pRb (1:25, overnight 4ºC)	NR	NR	0 (0.00)
Shah *et al.* (2007)	India	English	Retrospective cohort (2000-2003)	NR	60	M=54 (90.00) F=6 (10.00) (range= 18-75)	53 (88.33)	NR	Buccal mucosa: 60	Oral leukoplakia Submucous fibrosis (NR)	Hyperplasia Epithelial dysplasia (NR)	NR	IHQ	Rb1 (1:40, overnight 4ºC)	Nuclear	10	54 (90.00)
Soni *et al.* (2005)	India	English	Prospective cohort (NR)	(min: >24; max: NR)	90	M= 72 (80.00) F= 18 (20.00) (<35 years =37; ≥35years =53)	NR	NR	Buccal mucosa: 64 Tongue: 14 Gingiva: 3 Lip: 5 Others: 4	Oral leukoplakia (NR)	Hyperplasia Epithelial dysplasia (NR)	NR	IHQ	IF8 (1:100, NR)	Nuclear	10	30 (33.33)
Ghazali *et al.* (2003)	Malaysia	English	Retrospective cohort (NR)	56.4	9	M= 2 (22.23) F= 7 (77.77) Mean=61.66	4 (44.44)	1 (11.11)	Tongue: 2 Others:1 Multifocal:6	Proliferative verrucous leukoplakia** (Hansen criteria)	Verrucous hyperplasia Epithelial dysplasia (Batsakis criteria)	7	IHQ	NR (1:50, overnight 4ºC)	Nuclear	NR	7 (77.78)
Schoelch *et al.* (1999)*	USA	English	Retrospective cohort (NR)	NR	18	NR (NR)	NR	NR	NR	NR	Keratosis Epithelial dysplasia (NR)	16	IHQ	Rb1 (1:50, 1h, NR)	Nuclear	1	0 (0.00)
Girod *et al.* (1998)	Germany	English	Retrospective cohort (NR)	NR	113 (1 missing)	NR (NR)	NR	NR	NR	Oral leukoplakia Oral lichen planus (DÖSAK guidelines)	Keratosis Hyperplasia Epithelial dysplasia (Gräbel-Pietrusky and Hornstein criteria; Burkhardt criteria)	6	IHQ	Ab-1 (NR)	Nuclear	1	8 (70.00)

Abbreviations: n, number; m, months; y, years; SD, standard deviation; NR, not reported; IHQ, immunohistochemistry; OSCC, oral squamous cell carcinoma; DÖSAK guidelines: German-Austrian-Swiss Association for Head and Neck Tumours.

*The quantitative and qualitative datasets were additionally extracted from a second source (Schoelch ML, Le QT, Silverman S Jr, McMillan A, Dekker NP, Fu KK, Ziober BL, Regezi JA. Apoptosis-associated proteins and the development of oral squamous cell carcinoma. Oral Oncol. 1999 Jan;35(1):77-85).
**Two cases of patients with proliferative verrucous leukoplakia were also associated with oral submucous fibrosis.

3. Meta-analyses on the association between the loss of pRb expression and the malignant transformation risk of OPMDs

3.1 Subgroup analysis by geographical region

Figure S1. Forest plot graphically representing the stratified meta-analysis on the association between the loss of pRb expression and the malignant transformation risk of OPMDs by geographical region.

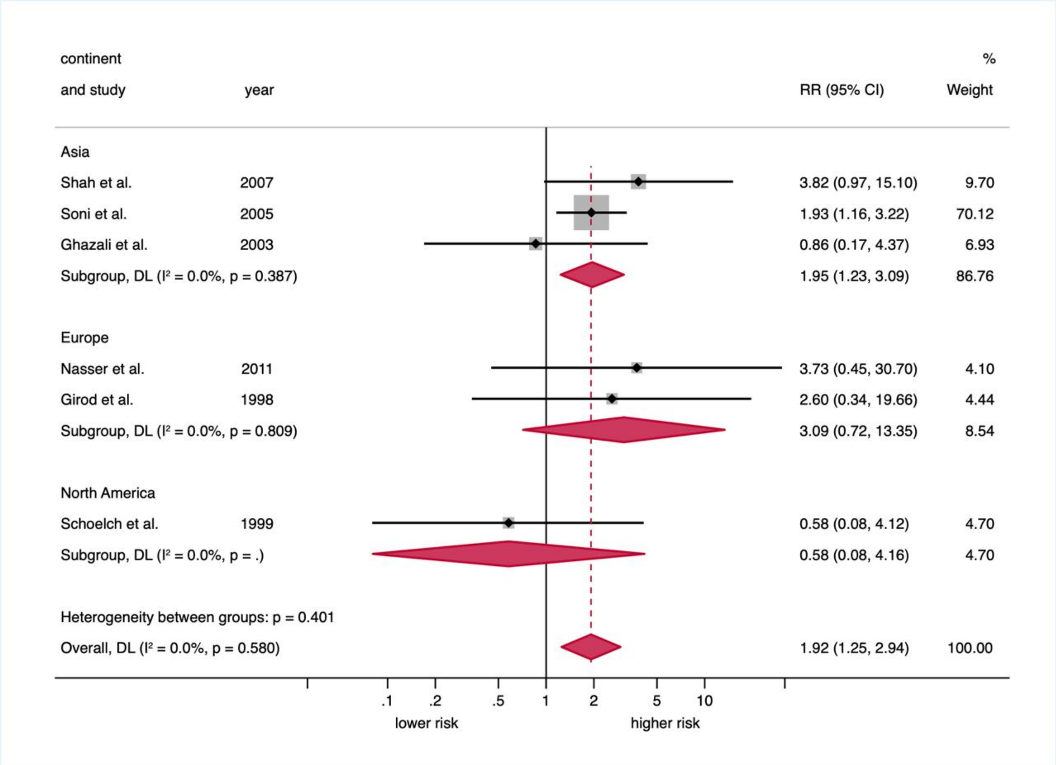

RR, relative risk; CI, confidence intervals, DerSimonian and Laird, DL. Random-effects model, inverse-variance weighting based on the DL method. A RR > 1 suggests that the loss of pRb expression is associated with a higher malignant transformation risk. Diamonds indicate the pooled RR with their corresponding 95% CIs.

3.2 Subgroup analysis by type diagnostic criteria

Figure S2. Forest plot graphically representing the stratified meta-analysis by type of diagnostic criteria (i.e., clinical lesions vs histopathological lesions).

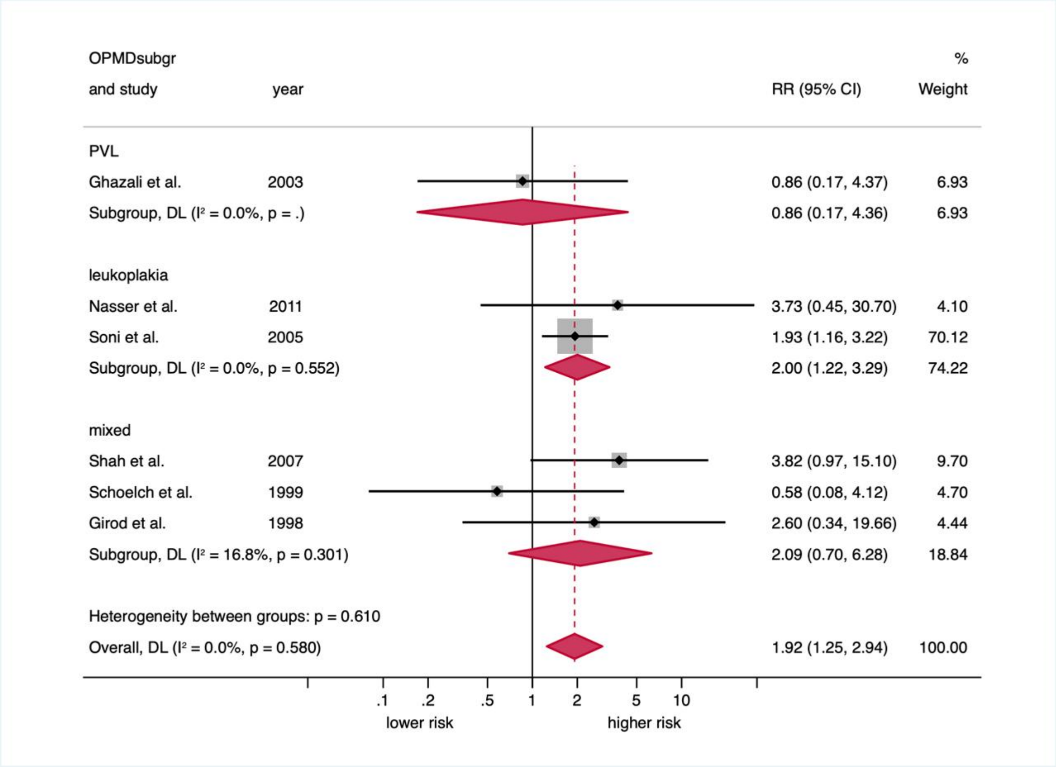

RR, relative risk; CI, confidence intervals, DerSimonian and Laird, DL; Random-effects model, inverse-variance weighting based on the DL method. A RR > 1 suggests that the loss of pRb expression is associated with a higher malignant transformation risk. Diamonds indicate the pooled RR with their corresponding 95% CIs.

3.3 Subgroup analysis by immunohistochemical pattern

Figure S3. Forest plot graphically representing the stratified meta-analysis on the association between the loss of pRb expression and the malignant transformation risk of OPMDs by immunohistochemical pattern.

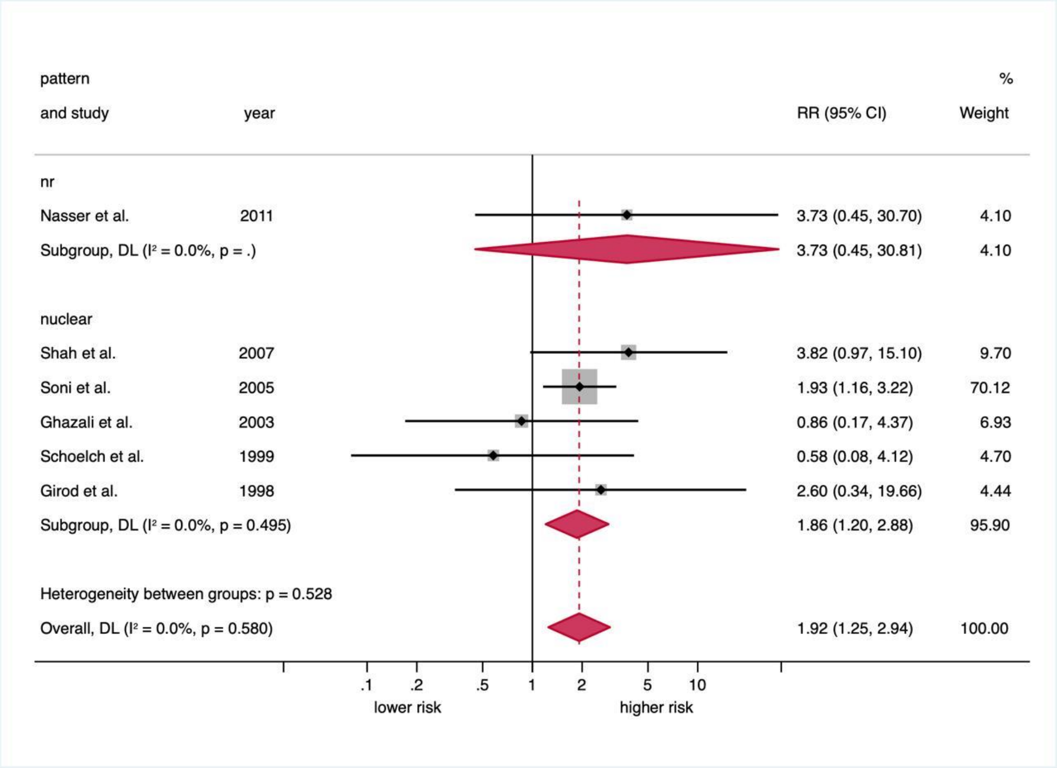

RR, relative risk; CI, confidence intervals, DerSimonian and Laird, DL; nr, not reported. Random-effects model, inverse-variance weighting based on the DL method. A RR > 1 suggests that the loss of pRb expression is associated with a higher malignant transformation risk. Diamonds indicate the pooled RR with their corresponding 95% CIs.

3.4 Subgroup analysis by anti-pRb antibody

Figure S4. Forest plot graphically representing the stratified meta-analysis on the association between the loss of pRb expression and the malignant transformation risk of OPMDs by anti-pRb antibody.

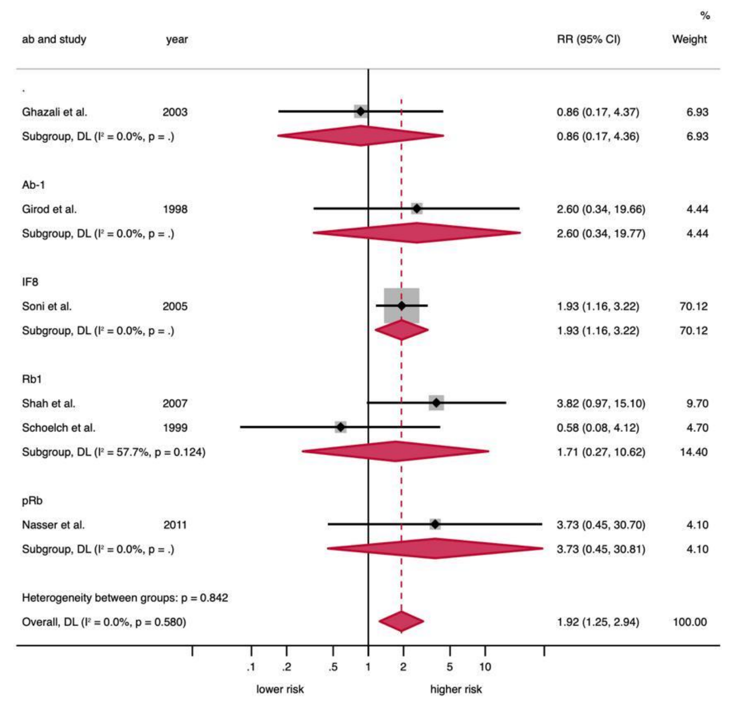

RR, relative risk; CI, confidence intervals, DerSimonian and Laird, DL. Random-effects model, inverse-variance weighting based on the DL method; nr, not reported. A RR > 1 suggests that the loss of pRb expression is associated with a higher malignant transformation risk. Diamonds indicate the pooled RR with their corresponding 95% CIs.

3.5 Subgroup analysis by anti-pRb antibody dilution

Figure S5. Forest plot graphically representing the stratified meta-analysis on the association between the loss of pRb expression and the malignant transformation risk of OPMDs by anti-pRb antibody dilution.

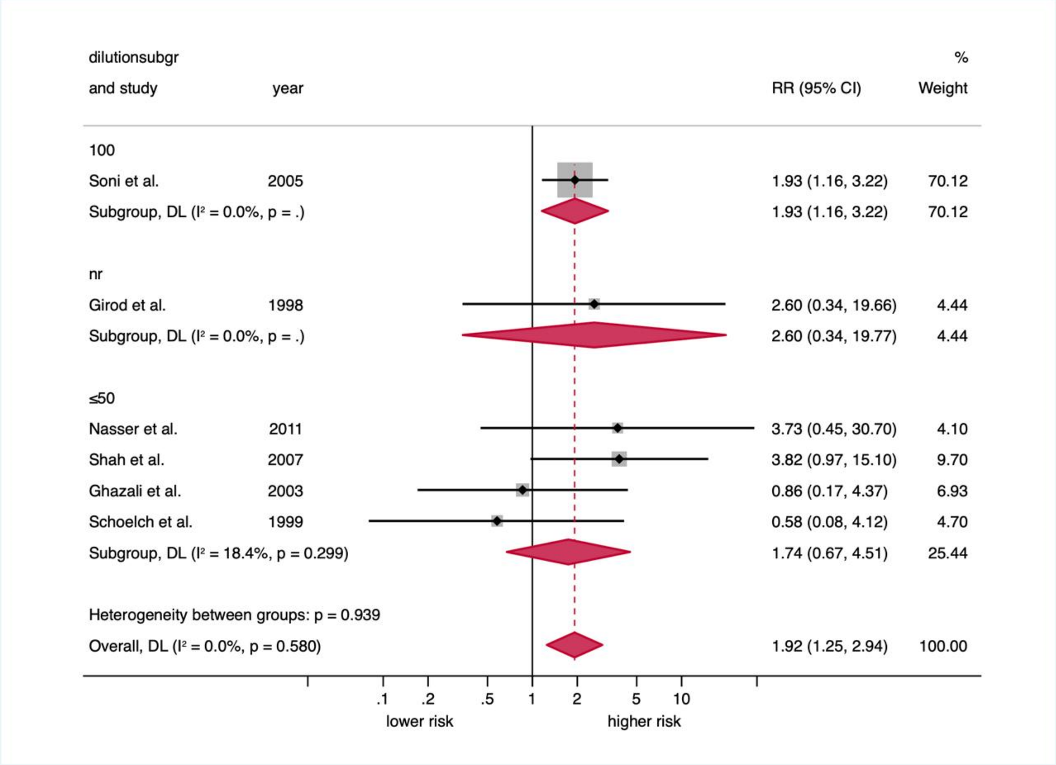

RR, relative risk; CI, confidence intervals, DerSimonian and Laird, DL; nr, not reported. Random-effects model, inverse-variance weighting based on the DL method. A RR > 1 suggests that the loss of pRb expression is associated with a higher malignant transformation risk. Diamonds indicate the pooled RR with their corresponding 95% CIs.

3.6 Subgroup analysis by anti-pRb antibody incubation time

Figure S6. Forest plot graphically representing the stratified meta-analysis on the association between the loss of pRb expression and the malignant transformation risk of OPMDs by anti-pRb antibody incubation time.

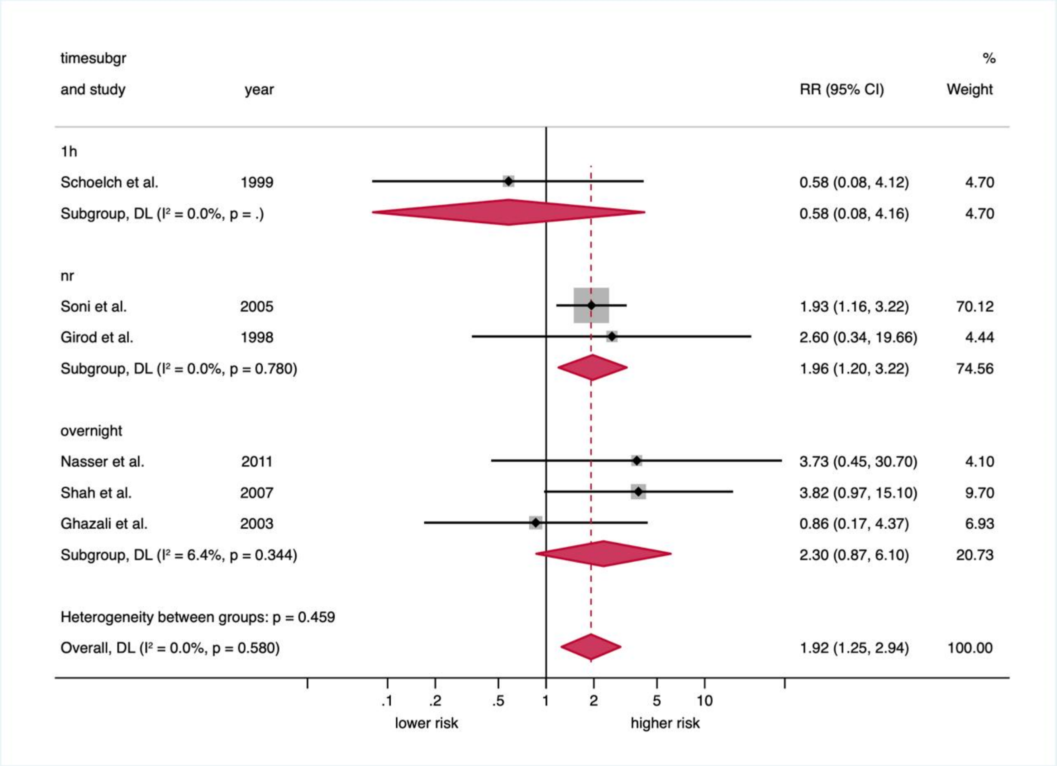

RR, relative risk; CI, confidence intervals, DerSimonian and Laird, DL; nr, not reported. Random-effects model, inverse-variance weighting based on the DL method. A RR > 1 suggests that the loss of pRb expression is associated with a higher malignant transformation risk. Diamonds indicate the pooled RR with their corresponding 95% CIs.

3.7 Subgroup analysis by anti-pRb antibody incubation temperature

Figure S7. Forest plot graphically representing the stratified meta-analysis on the association between the loss of pRb expression and the malignant transformation risk of OPMDs by anti-pRb antibody incubation temperature.

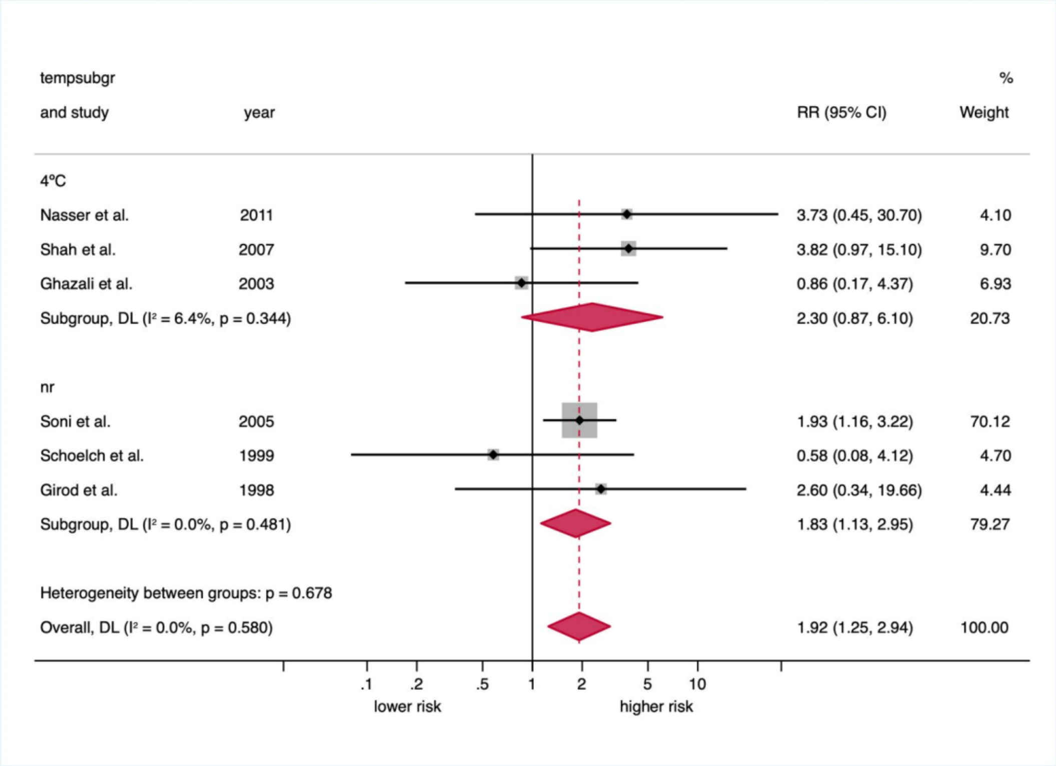

RR, relative risk; CI, confidence intervals, DerSimonian and Laird, DL; nr, not reported. Random-effects model, inverse-variance weighting based on the DL method. A RR > 1 suggests that the loss of pRb expression is associated with a higher malignant transformation risk. Diamonds indicate the pooled RR with their corresponding 95% CIs.

3.8 Subgroup analysis by cutoff point for pRb overexpression

Figure S8. Forest plot graphically representing the stratified meta-analysis on the association between the loss of pRb expression and the malignant transformation risk of OPMDs by cutoff point for pRb overexpression.

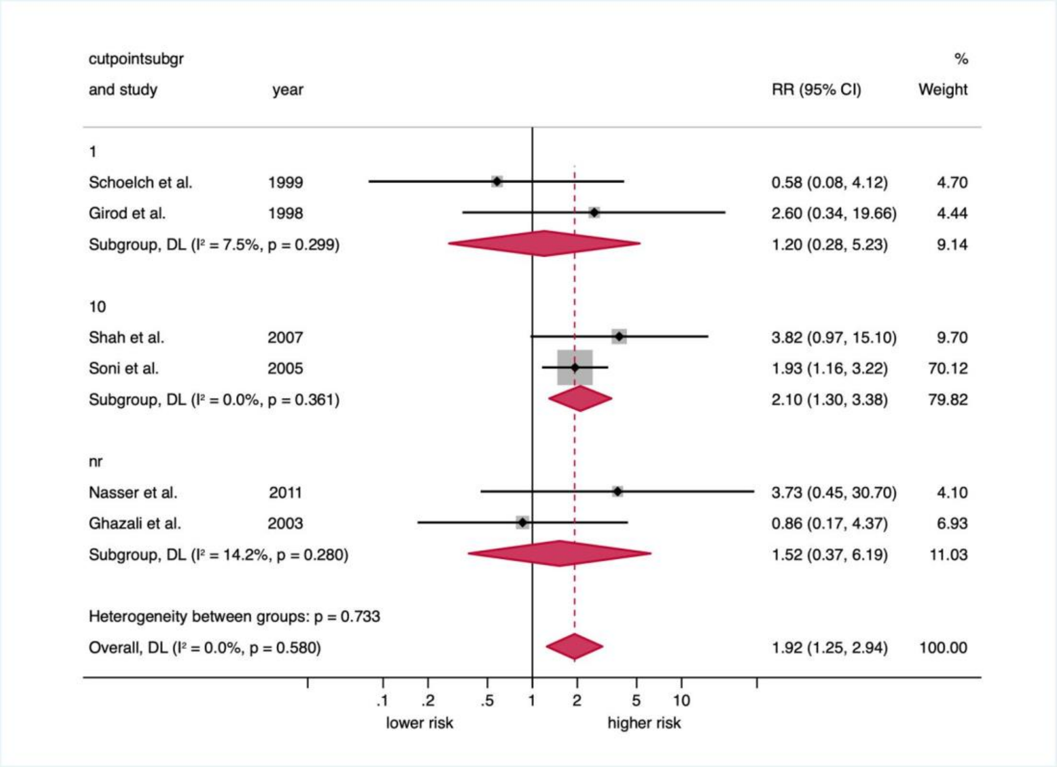

RR, relative risk; CI, confidence intervals, DerSimonian and Laird, DL; nr, not reported. Random-effects model, inverse-variance weighting based on the DL method. A RR > 1 suggests that the loss of pRb expression is associated with a higher malignant transformation risk. Diamonds indicate the pooled RR with their corresponding 95% CIs.

3.9 Subgroup analysis by overall risk of bias in primary-level studies

Figure S9. Forest plot graphically representing the stratified meta-analysis on the association between the loss of pRb expression and the malignant transformation risk of OPMDs by overall risk of bias in primary-level studies.

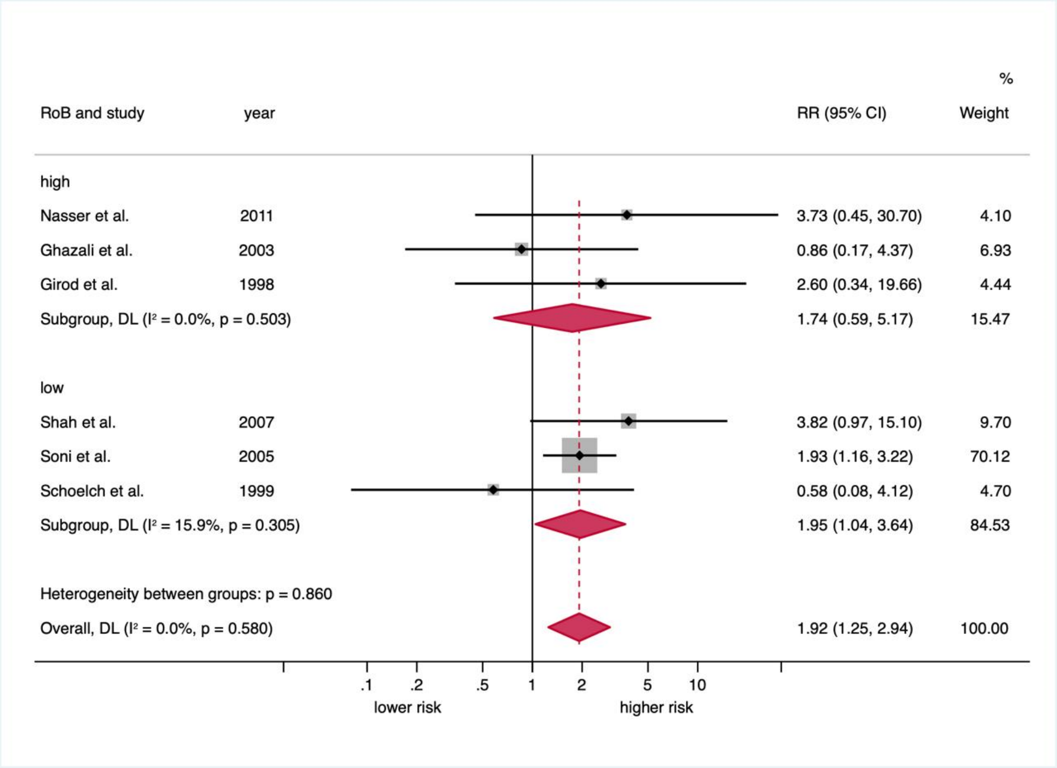

RR, relative risk; CI, confidence intervals, DerSimonian and Laird, DL. Random-effects model, inverse-variance weighting based on the DL method. A RR > 1 suggests that the loss of pRb expression is associated with a higher malignant transformation risk. Diamonds indicate the pooled RR with their corresponding 95% CIs.

4. Analysis of small-study effects

Figure S10. A funnel plot of estimated logRRs against their standard errors, graphically representing the analysis of small-study effects on the association between the loss of pRb expression and OPMDs malignant transformation risk.

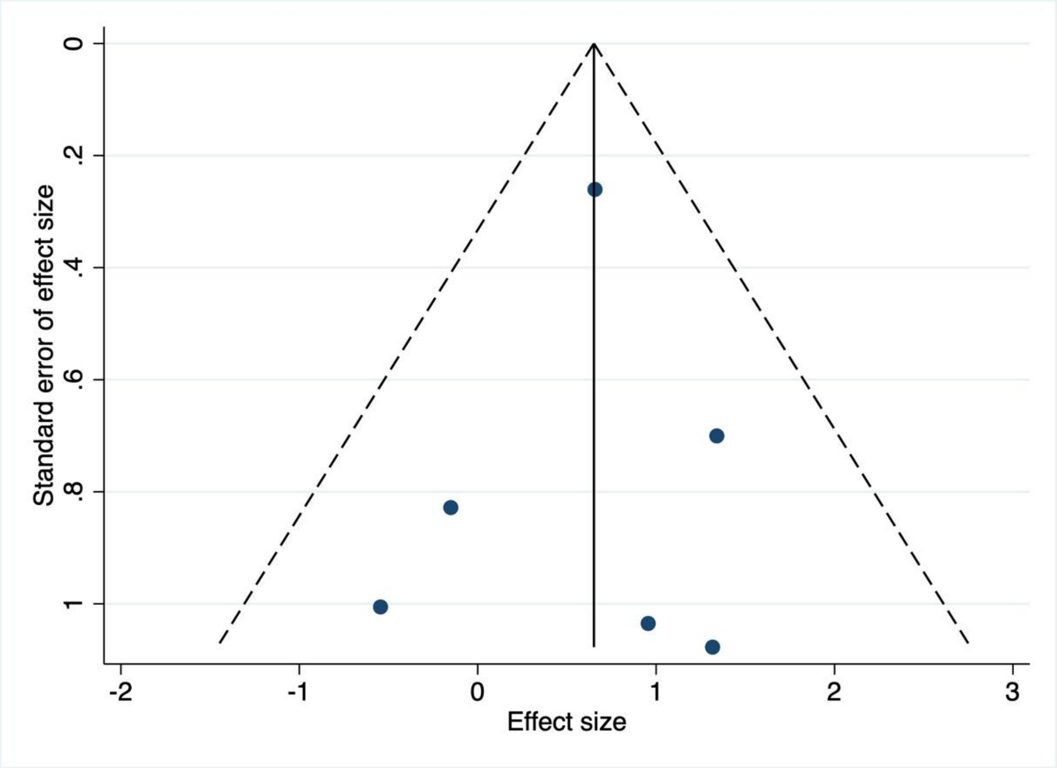

SE, standard error; RR, relative risk; log, natural logarithm (i.e., log base e). The black vertical line corresponds to the pooled effect size estimated in the meta-analysis. The two diagonal intermittent lines represent the pseudo-95% confidence interval. The blue circles represent the estimates from primary-level studies.

5. Sensitivity analysis (leave-one-out method).

Figure S11. Interval plot graphically representing the sensitivity analysis of the studies pooled in the meta-analysis on the association between the loss of pRb expression and OPMDs malignant transformation risk.

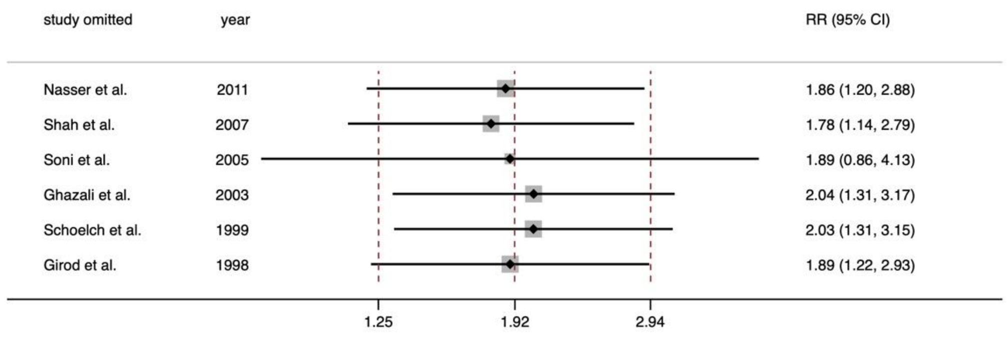

RR, relative risk; CI, confidence intervals. Sensitivity analysis ("leave-one-out" method) of the meta-analysis results, sequentially omitting one study at a time to investigate its influence on the overall result. In the interval plot, the usual diamond shape representing the usual pooled effect was replaced by vertical

6. List S1. List of full-text excluded studies, with reasons

Cross-sectional (n = 5)

Bascones-Martínez A, López-Durán M, Cano-Sánchez J, et al. Differences in the expression of five senescence markers in oral cancer, oral leukoplakia and control samples in humans. *Oncol Lett*. 2012;3(6):1319-1325. doi:10.3892/ol.2012.649

de Oliveira MG, Ramalho LM, Gaião L, Pozza DH, de Mello RA. Retinoblastoma and p53 protein expression in pre-malignant oral lesions and oral squamous cell carcinoma. *Mol Med Rep*. 2012;6(1):163-166. doi:10.3892/mmr.2012.876

Nakahara Y, Shintani S, Mihara M, Kiyota A, Ueyama Y, Matsumura T. Alterations of Rb, p16(INK4A) and cyclin D1 in the tumorigenesis of oral squamous cell carcinomas. *Cancer Lett*. 2000;160(1):3-8. doi:10.1016/s0304-3835(00)00546-2

Pande P, Mathur M, Shukla NK, Ralhan R. pRb and p16 protein alterations in human oral tumorigenesis. *Oral Oncol*. 1998;34(5):396-403. doi:10.1016/s1368-8375(98)00024-4

Thomas S, Balan A, Balaram P. The expression of retinoblastoma tumor suppressor protein in oral cancers and precancers: A clinicopathological study. *Dent Res J (Isfahan)*. 2015;12(4):307-314. doi:10.4103/1735-3327.161427

Not OPMD (n = 2)

Liu CJ, Chang KW, Chao SY, et al. The molecular markers for prognostic evaluation of areca-associated buccal squamous cell carcinoma. *J Oral Pathol Med*. 2004;33(6):327-334. doi:10.1111/j.1600-0714.2004.00092.x

Xu J, Gimenez-Conti IB, Cunningham JE, et al. Alterations of p53, cyclin D1, Rb, and H-ras in human oral carcinomas related to tobacco use. *Cancer*. 1998;83(2):204-212. doi:10.1002/(sici)1097-0142(19980715)83:2<204::aid-cncr2>3.0.co;2-q

Not immunohistochemistry (n = 1)

Rohatgi N, Kaur J, Srivastava A, Ralhan R. Smokeless tobacco (khaini) extracts modulate gene expression in epithelial cell culture from an oral hyperplasia. *Oral Oncol*. 2005;41(8):806-820. doi:10.1016/j.oraloncology.2005.04.010

Overlapping population (n = 1)

Shah NG, Trivedi TI, Tankshali RA, et al. Prognostic significance of molecular markers in oral squamous cell carcinoma: a multivariate analysis. *Head Neck*. 2009;31(12):1544-1556. doi:10.1002/hed.21126

Off topic (n = 1)

Flaitz CM, Hicks MJ. Molecular piracy: the viral link to carcinogenesis. *Oral Oncol*. 1998;34(6):448-453. doi:10.1016/s1368-8375(98)00057-8
